# Viscount Hill's and Dr. Giles's Speeches

**Published:** 1913-06-21

**Authors:** 


### Viscount Hill's and Dr. Giles's Speeches.

The Right Hon. Viscount Hill, in replying, said: Arising out of the speech of Sir Henry Burdett, I desire, on behalf of the hospital, to express to you, Sir, our sincere gratitude for and appreciation of the very kind and sympathetic way in which you have spoken, and for the detailed account which you have given us in proposing this toast, as well as for the very earnest appeal which you have made, not only to our friends gathered about us to-night, but an appeal which I feel sure will reach the most hard-hearted of those who will read it to-morrow morning. The hospital, as you have said, nine years ago was considered one of our youngest institutions. But I think you will agree with me to-night, ladies and gentlemen, that after the remarks, and the very detailed account, which have just been before us, I can claim for this hospital, as far as efficiency and economic management go, an equal position with any other London hospital. It is evident, from the history we have just listened to, that one great feature of this hospital has been the rapid growth of its work since the change from its old character as a home in which to train nurses, to one which led it to be numbered among the general hospitals of London in the year 1898. You have spoken, Sir, of the history of the hospital, and what it has done. May I say here that the success of this hospital has been greatly brought about by the energy and generosity of the late Sir Francis Cory-Wright and of the late Mr. Penny, two names which I venture to claim will always be remembered in connection with the growth of this hospital. And yet our success does not end there with our friends. There is another name which I think we must not overlook, namely, that of Mr. John Morley, to whom, I believe, we are indebted for giving us that admirable site upon which the hospital now stands, with its four and a half acres of open space, which is most valuable to our patients, and a great benefit to our staff. And he paid for it out of his own pocket. Then I must not overlook the fact that we appreciate, and are greatly indebted for, the treatment extended to us by the King's Fund. It is a great asset, and one that is very much appreciated by the management. We have also another old friend whom I must not overlook, namely, Sir Ernest Cassel, for his gift of £1,500. I would also like to mention a number of generous contributions which have been received from anonymous friends through "J. B. L." In your remarks, Sir, you alluded to our festival dinner of 1904. That was the first Festival which this hospital gave, and we have not held one since. On that occasion we had the task of raising £25,000 for extensions. You alluded to those extensions, and I am glad to say they have been completed, and one wing was paid for by the Worshipful Company of Drapers. At that dinner we realised over £4,000—a record collection for a first festival dinner. If we could only realise that amount to-night, what happiness it would bring to those who are responsible for the management of this hospital! That noble gift of the Drapers' Company enabled the Committee to carry out extensions which were opened on May 7, 1907, by our present King and Queen, accompanied by our Royal President. Here may I express how much the management appreciate the great interest which Her Royal Highness takes in this hospital, and may I be permitted to read a letter which was sent to me this morning?—for it shows how very much interest Her Royal Highness still takes in our hospital. It is from her private secretary : "Dear Lord Hill,—Her Royal Highness Princess Louise wishes me to write and say how pleased she is to hear that the Prince of Wales's Hospital is getting on so well. The hospital is admirably situated, and she knows how well managed it is, and the number of people who benefit by its presence in their midst. For these reasons she trusts that the people will realise how necessary it is to support so deserving an institution, which is doing such admirable work. She is very much gratified to hear about the Convalescent Home at Nazing, which she understands has been put up chiefly owing to the generosity of Mr. Hargreaves, and furnished at Mr. Lebus's expense. She feels the deepest thanks of all connected with the hospital are due to those two gentlemen for their generosity. I am to assure you that Princess Louise continues to take the keenest interest in the hospital and its work, and Her Royal Highness hopes the dinner to-night will be in every way a success, and that the hospital will benefit thereby.—Yours sincerely, GEORGE LEIGH, Secretary." You alluded, Sir, to the medical profession. I should like to say that we have an excellent medical staff. I may say that it is second to none in London. The hospital has certainly gained a great reputation by the eminence of its medical staff, and the wonderful work carried on, both surgical and medical, by these gentlemen who have given their services for so many years, voluntarily, for this charity. Personally I find it difficult, and almost impossible, to find words to express to those gentlemen our high appreciation of their valuable services—services which they still continue to render to the patients under their charge at this hospital. The management has certainly received commendation from quarters best able, and most qualified, to judge upon such matters; they consider that this is the most economically worked hospital in London, and this is done without in any way impairing the efficiency of our institution or hampering its work. The principle has always been to spend the money committed to our charge for the specific purpose for which it was given, namely, the nursing and relief of the sick and suffering. The secret of our successes has always been the avoidance of waste and unnecessary expenditure. So much for the past history of the hospital. I should now like to say a few words as to its future. We have had a most earnest appeal from Sir Henry Burdett, but there is one important fact which must not be omitted on this occasion. I refer to the presentation of the Convalescent Home at Nazing. This will be no charge upon the Hospital Fund; the Home is self-sufficient, and ought to be of the greatest value to this hospital, and yet not too large a burden. The idea of this Convalescent Home originated at a drawing-room meeting held at Mrs. Smith-Bosanquet's, who is the wife of one of the members of the governing body, and who I am glad to see present with us to-night. It is through his energy that it has been brought to a successful issue, by the assistance of the generosity and the energy of Mr. W. E. Hargreaves, who I am also glad is with us to-night. We have still some more friends for our future. The furniture for this Home has been very kindly presented by Mr. and Mrs. Hermann Lebus. Both are with us to-night. And I should like here to express how very grateful we are to Mr. Lebus, and I think I may couple with that the name of his brother, Mr. Lewis Lebus, who is joining in this beneficent donation. The Convalescent Home, I believe, is ready for occupation. I also desire to thank all those generous friends in the district of Broxbourne and Hoddesdon who have in any way assisted in bringing and securing this nice little Convalescent Home for the use of this hospital. I understand also that many of our generous friends in those districts have guaranteed the annual cost of the beds, and I would like here to most cordially thank them. We are further indebted to the *Daily Express* Radium Fund for a cheque for £275 for radium, which is to be entirely set apart for the purpose of supplying that valuable metal for the purpose of treatment. I do not intend to make a long speech. We have heard all the details so eloquently put forth by Sir Henry Burdett, who knows the hospital much better than I do, or can ever hope to do, although I confess I take an extreme interest in it. Later I shall ask Mr. Drew to announce the amount of the donations, and you will find a few vacant lines on the cards in front of you, which I hope you will fill up.

In conclusion, I would inform this gathering to-night that this is no cottage hospital. The Prince of Wales's Hospital is a general hospital for North and North-East Middlesex, Essex, and Hertfordshire. We are always very hard at work, and we are overcrowded, as you have already heard, and we have to refuse many patients because of the accommodation being too limited. For that reason we are appealing to you to-night to endeavour to raise a fund to meet this extra need of accommodation in the district. I have to thank you again, Sir Henry Burdett, for your eloquent speech, and for your very sympathetic remarks; and I thank you, ladies and gentlemen, for your indulgence in listening to me, and I thank you also, on behalf of the Governors, for the warm reception which you have given this toast. Before I resume my seat I would like to say I have a letter from Mr. Walton, who lives at Hoddesdon, and who is much interested in the hospital. He has been endeavouring to get our labouring classes to understand the needs of the hospital, and the necessity for supporting it. He held a *fête* on Whit Monday, and the result of that, which he managed, and in which he persuaded the working men of the district to help, so that it worked admirably, and the men worked enthusiastically, was that he has been able to send to our Treasurer a cheque for £80. The weather was wet, and so there was not a very good attendance, but the receipts were £100 12s. 7d., and Mr. Walton is leaving in the Bank £2 9s. to carry forward for next year, and his expenses were £17 10s. I think that is a very worthy effort.

